# Supplementary material for: In Silico Characterization of Calcineurin from Pathogenic Obligate Intracellular Trypanosomatids: Potential New Biological Roles
Source: Biomolecules. 2021 Sep 7;11(9):1322. doi: 10.3390/biom11091322 (PMC8470620; doi:10.3390/biom11091322)
Supplement: Supplementary file 1 [file biomolecules-11-01322-s001.zip › biomolecules-1241702-supplementary.pdf]

Table S1. ScanProsite Score hits by 1 profile (pattern PS00018*)

Loop	*Hs*CaNB-1	*Tc*CaNB	*Lspp*CaNB
1	8181	None	9855
2	12840	8544	9018
3	14402	13202	13621
4	13286	10915	12589

*EF-hand calcium-binding domain.

Table S2. Myristoylator results

*Hs*CaNB-1 = The protein is predicted as myristoylated			
	Positive	Negative	Score
Average response of 25 neural networks	0.994907	0.00506406	0.98984294
*Tc*CaNB = The protein is predicted as non-myristoylated			
	Positive	Negative	Score
Average response of 25 neural networks	0.426957	0.573101	-0.146144

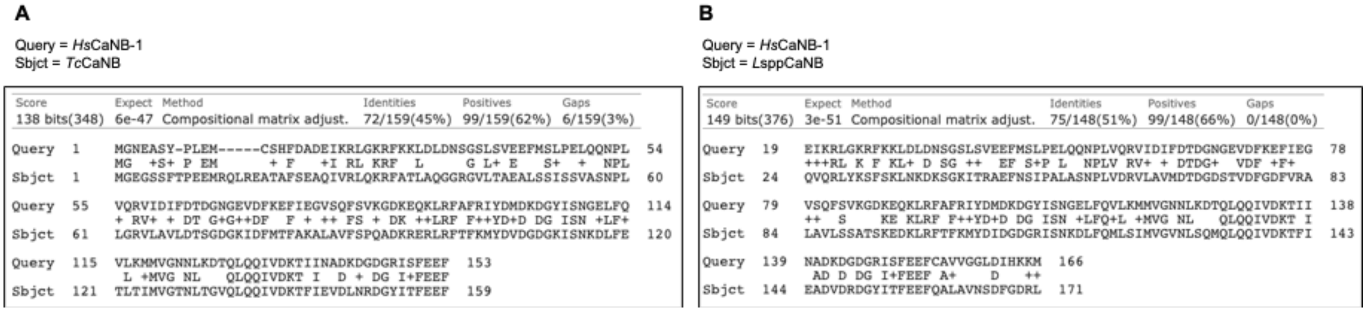

Figure S1. BLAST protein comparison of *Hs*CaNB-1 with *Tc*CaNB (A) and *Hs*CaNB-1with *L*sppCaNB (B) using Blast 2 sequences interface (https://blast.ncbi.nlm.nih.gov/Blast.cgi?PROGRAM=blastp&PAGE_TYPE=BlastSearch&BLAST_SPEC=blast2seq&LINK_LOC=blasttab&LAST_PAGE=blastp&BLAST_INIT=blast2seq)

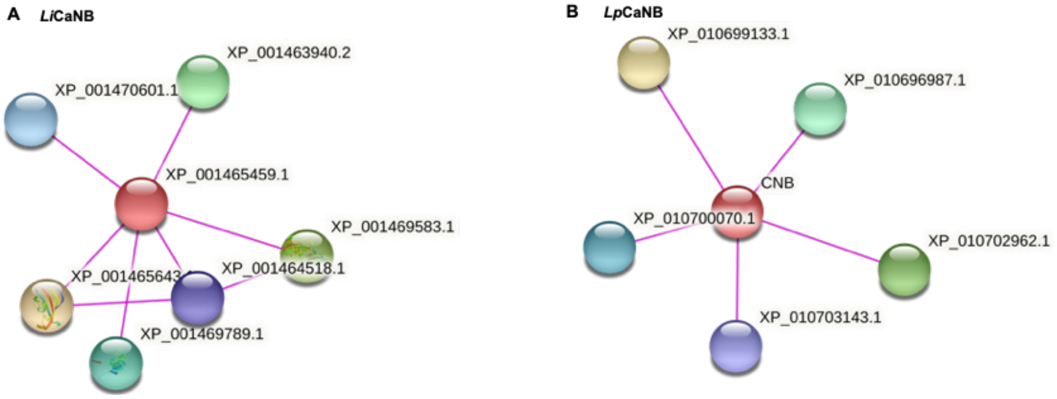

Figure S2. Protein-protein interaction network of the *Leishmania infantum* (*Li*CaNB) and *Leishmania panamensis* (*Lp*CaNB) CaNB proteins (STRING V.11.0). The line indicates the type of interaction evidence (experimentally determinated). Parameters: Score (high confidence 0.7), sources of interaction used: experimental and databases. In A: XP_001465459.1 (Calcineurin B subunit, putative), XP_001470601.1 (Serine/threonine protein phosphatase, putative), XP_001463940.2 (Uncharacterized protein), XP_001465643.1 (Peptidylprolyl isomerase, Fk506-binding protein 1-like protein), XP_001469789.1 (Serine/threonine protein phosphatase 2B catalytic subunit A2, putative), XP_001464518.1 (Uncharacterized protein) and in B: CNB (Calcineurin B subunit, putative), XP_010699133.1 (Peptidylprolyl isomerase), XP_010696987.1 (Serine/threonine-specific protein phosphatase, putative), XP_010700070.1 (Serine/threonine-protein phosphatase), XP_010703143.1 (Serine/threonine protein phosphatase 2B catalytic subunit A2, putative) and XP_010702962.1 (Peptidylprolyl isomerase).

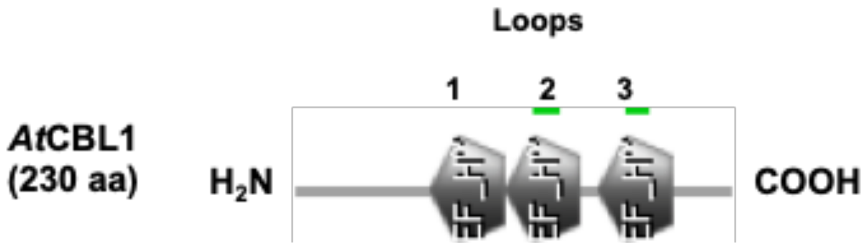

Figure S3. Protein domain architecture of calcineurin B-like of *Arabidopsis thaliana* (*At*CBL1)
